# Soluble Ecto-5′-nucleotidase (5′-NT), Alkaline Phosphatase, and Adenosine Deaminase (ADA1) Activities in Neonatal Blood Favor Elevated Extracellular Adenosine* [Fn FN2]

**DOI:** 10.1074/jbc.M113.484212

**Published:** 2013-07-29

**Authors:** Matthew Pettengill, Simon Robson, Megan Tresenriter, José Luis Millán, Anny Usheva, Taiese Bingham, Mirjam Belderbos, Ilana Bergelson, Sarah Burl, Beate Kampmann, Laura Gelinas, Tobias Kollmann, Louis Bont, Ofer Levy

**Affiliations:** From the ‡ Department of Medicine, Division of Infectious Diseases, Boston Children's Hospital, Boston, Massachusetts 02115,; the ¶ Department of Medicine, Beth Israel Deaconess Medical Center, Boston, Massachusetts 02215,; § Harvard Medical School, Boston, Massachusetts 02115,; the ‖ Sanford Children's Health Research Center, Sanford-Burnham Medical Research Institute, La Jolla, California 92037,; the ** Department of Pediatrics, University Medical Center Utrecht, 3584 CX Utrecht, The Netherlands,; the ‡‡ Vaccinology Theme Group, Medical Research Council Unit, Fajara, The Gambia, and; the §§ Department of Pediatrics, Imperial College London, London W2 IPG, United Kingdom, and; the ¶¶ Experimental Medicine Program, Department of Medicine, and; the ‖‖ Division of Infectious and Immunologic Diseases, Department of Pediatrics, University of British Columbia, Vancouver, British Columbia V6T 1Z4, Canada

**Keywords:** Adenosine, Adenosine Receptor, ADP, AMP, ATP, Immunology, Infectious Diseases, Innate Immunity, Purine, Purinergic Agonists

## Abstract

Extracellular adenosine, a key regulator of physiology and immune cell function that is found at elevated levels in neonatal blood, is generated by phosphohydrolysis of adenine nucleotides released from cells and catabolized by deamination to inosine. Generation of adenosine monophosphate (AMP) in blood is driven by cell-associated enzymes, whereas conversion of AMP to adenosine is largely mediated by soluble enzymes. The identities of the enzymes responsible for these activities in whole blood of neonates have been defined in this study and contrasted to adult blood. We demonstrate that soluble 5′-nucleotidase (5′-NT) and alkaline phosphatase (AP) mediate conversion of AMP to adenosine, whereas soluble adenosine deaminase (ADA) catabolizes adenosine to inosine. Newborn blood plasma demonstrates substantially higher adenosine-generating 5′-NT and AP activity and lower adenosine-metabolizing ADA activity than adult plasma. In addition to a role in soluble purine metabolism, abundant AP expressed on the surface of circulating neonatal neutrophils is the dominant AMPase on these cells. Plasma samples from infant observational cohorts reveal a relative plasma ADA deficiency at birth, followed by a gradual maturation of plasma ADA through infancy. The robust adenosine-generating capacity of neonates appears functionally relevant because supplementation with AMP inhibited whereas selective pharmacologic inhibition of 5′-NT enhanced Toll-like receptor-mediated TNF-α production in neonatal whole blood. Overall, we have characterized previously unrecognized age-dependent expression patterns of plasma purine-metabolizing enzymes that result in elevated plasma concentrations of anti-inflammatory adenosine in newborns. Targeted manipulation of purine-metabolizing enzymes may benefit this vulnerable population.

## Teaser

**Background:** Newborns have elevated plasma adenosine levels, which may influence their immunological function.

**Results:** Compared with adults, newborns have elevated plasma 5′-NT and alkaline phosphatase activities and lower adenosine deaminase activity.

**Conclusion:** Soluble enzymes significantly influence extracellular purine metabolism in blood, and the levels of these enzymes in newborns promote elevated adenosine.

**Significance:** Higher adenosine generation in newborn blood may promote an anti-inflammatory immunological status.

## Introduction

Purine signaling nucleotides and nucleosides, primarily adenosine triphosphate (ATP) and adenosine, are critical pathophysiologic mediators. In the context of immunity and inflammation, extracellular ATP (eATP)^6^ is important for T-cell activation (1, 2) and proliferation (3, 4), promoting neutrophil adhesion (to endothelial cells (5)) and degranulation (6, 7), reactive oxygen species production (8), and multiple other proinflammatory immune cell functions (9). Extracellular adenosine (eAdo) is primarily generated by the metabolism of eATP and has an opposing profile of immunoregulatory effects from the precursor molecule; eAdo inhibits neutrophil-endothelial adhesion (10, 11) and degranulation (12), reactive oxygen species production (13, 14), and macrophage production of proinflammatory/Th1-polarizing cytokines (IL-12p70, TNF-α) (9) as well as T-cell proliferation and effector functions (15, 16). eATP is primarily transported outside of cells by vesicular trafficking or secreted via pannexin-1 channels (17), where it can then stimulate P2 purinergic receptors and also serve as a source of eAdo through sequential dephosphorylation by several types of ectonucleotidases leading to P1 (Ado) receptor signaling (18). Thus, the enzymes involved in extracellular purine metabolism critically regulate whether eATP release results in largely P2 receptor-mediated proinflammatory sequelae or P1 receptor stimulation and an anti-inflammatory response.

There are four families of ectonucleotidases (19): ENTPDs (including ENTPD1 (CD39); substrates ATP and ADP), ectonucleotide pyrophosphatase/phosphodiesterases (substrates ATP and ADP), alkaline phosphatases (APs; substrates ATP, ADP, and AMP), and ecto-5′-nucleotidase (5′-NT (CD73); substrate AMP). Additionally, it was recently shown that adenylate kinase (AK1) influences extracellular adenine nucleotide pools via transphosphorylation (20). 5′-NT and AP are both attached to the plasma membrane by a glycosylphosphatidylinositol anchor and are found in plasma, possibly as a consequence of glycosylphosphatidylinositol-specific phospholipase activity (21–24). Of note, eAdo can be taken up into cells via nucleoside transporters (25) or be metabolized by adenosine deaminase (primarily ADA1, whereas the product of another gene (*CECR1*), referred to as ADA2, has little activity near physiological concentrations of Ado), generating inosine. Extracellular ADA1 is found either soluble in fluids or cell-associated via binding to CD26 (26) or Ado receptors (27). Increased ectonucleotidase activity would both diminish eATP exposure and enhance eAdo production, shifting subsequent cellular responses to an anti-inflammatory profile. In addition, modified levels of ADA1 activity or cellular adenosine transport could promote eAdo retention. An ectopurine enzyme profile that favors the metabolism of eATP and production and retention of eAdo should then mediate dampening of inflammatory and Th1-polarizing immune responses.

Innate immune responses in human newborns are distinct from those of adults (28), favoring higher production of Th2- and Th17-polarizing cytokines (*e.g.* IL-4 and IL-6) and diminished production of proinflammatory/Th1-polarizing cytokines (*e.g.* TNF-α and IL-12p70) in response to Toll-like receptor (TLR) agonists or whole microbes *in vitro* (29–31). Impaired neonatal production of key cytokines, such as TNF-α, may contribute to impaired mobilization and activation of phagocytes and to impaired vaccine responses (28). Interestingly, the polarized neonatal cytokine profile may be influenced by soluble factors in newborn plasma (29, 32, 33), which contains significantly higher levels of eAdo than adult plasma (32). In the current study, we assessed whether the difference in eAdo in newborn blood was due to differential levels of blood ectoenzyme expression. We found that direct eAdo-generating enzymes and ADA activities in whole blood were primarily soluble, not cell- or microparticle-associated. Remarkably, newborn whole blood *ex vivo* generated more and metabolized less eAdo than adult blood from exogenous purine precursors (ADP or AMP), due primarily to elevated soluble 5′-NT and tissue-nonspecific alkaline phosphatase (TNAP)-mediated AMPase activity and lower soluble ADA1 activity, respectively.

## MATERIALS AND METHODS

### 

#### 

##### Reagents

Adenosine 5′-(α,β-methylene)-diphosphate (APCP) was obtained from Sigma. EDTA and Hanks' buffered salt solution with (HBSS+) or without (HBSS−) calcium and magnesium were from Invitrogen. MLS-0038949 (34) was purchased from EMD Millipore (Billerica, MA). *erythro*-9-(2-Hydroxy-3-nonyl)adenine hydrochloride (EHNA) and 2,6-bis(diethanolamino)-4,8-dipiperidinopyrimido[5,4-*d*]pyrimidine (dipyridamole) were acquired from Tocris Cookson (Bristol, UK). Ficoll-Paque, Percoll, ammonium hydroxide, and 2-propanol were purchased from Fisher. 8-Aminoguanosine was obtained from Santa Cruz Biotechnology, Inc. Other reagents and sources are listed with assay descriptions.

##### Blood Collection

Peripheral blood was collected after informed consent from healthy adult volunteers according to Boston Children's Hospital Institutional Review Board-approved protocols (mean age 31.3 years), and newborn cord blood (mean gestational age 38.9 weeks) was collected immediately after elective cesarean section delivery (epidural anesthesia) of the placenta. Births to HIV-positive mothers were excluded. Human experimentation guidelines of the United States Department of Health and Human Services, the Brigham and Women's Hospital, Beth Israel Medical Center, and Boston Children's Hospital were observed, following protocols approved by the local institutional review boards. Number of repeats (*n*) indicates number of independent experiments. For primary human cells (or plasma), no subject was studied more than once in each of the different experiments. Blood was collected into syringes containing a final concentration of 20 units/ml heparin (Sagent Pharmaceuticals, Schaumberg, IL) and used within 2 h of collection.

##### Blood Plasma Collection from International Cohorts

Blood plasma was collected in a cross-sectional study (as described previously (35)) at birth and at 1, 2, 3, 4, 6, 9, and 12 months at the Medical Research Council Sukuta Health Centre in Fajara, The Gambia. Briefly, for every study subject, written informed consent of a parent/guardian was obtained. Children were excluded if they had any signs of intercurrent infection. Neonates were also excluded if they had a low birth weight (<2.5 kg) or were a twin. Venous blood was collected into tubes containing heparin at 7.5 units/ml blood and centrifuged at 1500 rpm for 10 min, and plasma was removed and frozen for future analysis. A separate longitudinal study at birth and at 1 and 2 years of age from which plasma was obtained was approved by the Institutional Ethics Review Board at both the University of Washington and the University of British Columbia, and details of this cohort and subject recruitment have been described previously (36). Briefly, following written and informed consent, cord or peripheral blood was collected via sterile venipuncture into tubes containing 143 USP units of sodium heparin (Vacutainer Tubes, BD Biosciences). Blood was centrifuged at 1250 rpm for 10 min, and plasma was removed and frozen at −80 ºC until further analysis.

##### Plasma and Hemocyte Preparation

Plasma was prepared sequentially by centrifuging whole blood at 500 × *g* for 5 min and carefully collecting the upper layer (platelet-rich plasma). Additional centrifugation at 3,000 × *g* for 30 min yielded platelet-poor plasma that could be spun at 16,000 × *g* for 30 min to collect microparticle-free plasma (MFP). Washed hemocytes were generated by centrifuging whole blood at 500 × *g* for 5 min and replacing the plasma volume with HBSS+, followed by two more cycles of centrifugation and supernatant replacement with HBSS+. All centrifugations were performed at ambient temperature (24 °C).

##### Neutrophil and Erythrocyte Isolation

Neutrophils were isolated as described previously (37). Briefly, whole blood was layered onto Hypaque-Ficoll and centrifuged at 1100 × *g* for 30 min at room temperature (brake off), and the peripheral blood mononuclear cell/cord blood mononuclear cell layer and liquid phase were carefully removed. The upper portion of the RBC/granulocyte layer was carefully collected and resuspended in PBS before 1:1 dilution with a 3% dextran (Pharmacosmos) solution (in saline) followed by repeated inversion (10 times) prior to allowing the cells to settle for 20 min at room temperature. The leukocyte-rich upper layer was added to new tubes for centrifugation at 500 × *g* for 10 min. Erythrocytes were lysed by resuspending the pellet in H_2_O for 25 s, followed by immediate tonicity restoration with 2× PBS. Cells were spun again at 500 × *g*, and the resulting pellet was resuspended in HBSS− and spun again at 500 × *g* for 10 min. The final pellet was resuspended in HBSS+, and cell density was determined by hemocytometer and adjusted as necessary with HBSS+ to a density of 4 × 10^6^ cells/ml (viability, as measured by trypan blue exclusion during quantitation, was >90% for each sample). Erythrocytes were collected from the remaining cell pellet following RBC/granulocyte layer removal from Hypaque-Ficoll separation and were washed three times with HBSS+ (with centrifugation at 500 × *g* for 5 min) before resuspension in an equal volume of HBSS+ (500 μl of RBC cell pellet + 500 μl of HBSS+). The purity of erythrocytes (RBCs) collected by this method was evaluated by the absence of leukocytes in Wright-stained smears. RBC density was also determined by hemocytometer.

##### Enzyme Assays and Thin Layer Chromatography

Enzymatic modification of nucleotide and nucleoside substrates was evaluated utilizing [^14^C]ADP (PerkinElmer Life Sciences), [^14^C]AMP, and [^14^C]adenosine (Moravek Biochemicals). Substrates were added to prewarmed samples at a concentration of 0.5, 5.0, 50, or 200 μm, as indicated, during gentle vortexing before incubating at 37 °C in a dry bath for specified times. The 50 μm substrate concentration was selected for the majority of the samples to model putative responses during inflammatory tissue damage, during which elevated levels of extracellular purine substrate may be present, and also for technical considerations to aid in the detection of metabolic products. Plasma enzyme activity measurements utilizing a saturating concentration of substrate (200 μm) and collection at multiple time points (times indicated) were used to calculate rates of activity in plasma and were within the linear range of activity. Samples with non-saturating levels of substrate are displayed as accumulated reaction product at the indicated time point. Reactions were terminated by adding 3 volumes of a solution containing 50 mm EDTA (Invitrogen) and 30 μm EHNA (ADA inhibitor; Tocris) in HBSS (Invitrogen). Controls for analytes of interest ([^14^C]adenosine or [^14^C]inosine; Moravek) were prepared at assay-appropriate concentration ranges, and controls and samples were then applied to silica gel matrix thin layer chromatography plates (Sigma-Aldrich) and migrated until the solvent (6:3:1, 2-propanol/ammonium hydroxide/distilled H_2_O) front reached 4 cm beyond the application site. Plates were dried and incubated in a storage phosphor screen cassette overnight, after which the screens were analyzed on a GE Storm 860 Imager. Analytes were quantified relative to controls by densitometry utilizing ImageJ software (version 1.43u; National Institutes of Health). For assays utilizing 0.5 μm [^14^C]adenosine substrate, plates were incubated in a storage phosphor screen cassette for 14 days before analysis.

##### Flow Cytometry

Whole blood was washed (2 ml of blood + 10 ml of HBSS+; centrifuged at 300 × *g* for 5 min; 10 ml supernatant removed and the remainder reconstituted) to remove the majority of plasma. Fluorochrome-conjugated monoclonal antibodies against CD3 (phycoerythrin-Cy7), CD19 (APC-Cy7), CD14 (Alexa Fluor 700), CD66b (PerCP Cy5.5), TNAP (phycoerythrin), and CD73/5′-NT (APC) (all from BD Biosciences); CD39/ENTPD1 (FITC; eBioscience); and ADA1 (Calbiochem; labeled in-house with Pacific Blue APEX antibody labeling kit per manufacturer's recommendations (Invitrogen), which was also performed for an isotype control antibody) or appropriate phycoerythrin-, APC-, and FITC-conjugated isotype control antibodies (BD Biosciences and eBiosciences) were added directly to 200 μl of washed whole blood at room temperature for 30 min. Samples were added to BD Facs lysing solution for 10 min and centrifuged at 500 × *g* for 5 min. Supernatants were removed, and cell pellets were washed with PBS and centrifuged again. Cell pellets were finally resuspended in 1% paraformaldehyde (VWR) and analyzed on a BD LSR II flow cytometer within 48 h. Data were collected uncompensated, and compensation, doublet exclusion, and data analysis were performed with FlowJo software (version 9.2; TreeStar).

##### ADA Chromogenic Assay

ADA1 and ADA2 activities in plasma samples were determined with an adenosine deaminase assay kit per the manufacturer's instructions (Diazyme Laboratories, Poway, CA), run in duplicate with or without EHNA (20 μm). ADA2 is not EHNA-sensitive; thus, activity in EHNA-containing wells was considered to reflect ADA2 activity. ADA1 activity was calculated by subtracting ADA2 activity from total ADA activity.

##### Alkaline Phosphatase Isoform Assay

Blood was collected as described previously, but without anti-coagulant, and left at ambient temperature until coagulation (up to 2 h). Serum samples were then sent to Mayo Medical Laboratories for evaluation of alkaline phosphatase isoform content by electrophoresis through alkaline-buffered agarose gels with or without wheat germ lectin for separation, essentially as described previously (38), followed by an enzymatic assay with a chromogenic substrate, 5-bromo-4-chloro-3-indolyl phosphate/nitro blue tetrazolium in aminomethyl propanol buffer, pH 10.0, utilizing the Sebia Hydragel 7 and 15 ISO-PAL systems (Sebia, Norcross, GA). Results were reported in units/liter for bone, liver type 1, and liver type 2 AP. Intestinal AP was detected in 1 adult sample (10 units/liter). No placental or germ cell AP was reported.

##### Whole Blood Stimulation with Staphylococcus epidermidis

Experiments assessing bacteria-induced cytokine production employed *S. epidermidis* 1457, a clinical strain from a patient with a central catheter infection that was previously isolated by Mack *et al.* (39). Anti-coagulated whole blood was incubated with APCP (an inhibitor of 5′-NT) or buffer control before stimulation with 10^7^ live *S. epidermidis*/ml, lipopolysaccharide (LPS; 1 μg/ml), or vehicle control for 4 h during end-over-end rotation at 37 °C. Supernatants were collected following centrifugation and stored at −80 °C prior to measurement of TNF-α, IL-6, IL-1β, IL-23, and IL-10 by ELISA per the manufacturer's recommendations (BD Biosciences for TNF-α and IL-10; eBioscience for IL-6, IL-23, and IL-1β).

Plasma alkaline phosphatase activity was evaluated in diethanolamine buffer with 1 mm Mg^2+^, 20 μm Zn^2+^ and 2.7 mm *p*-nitrophenyl phosphate (Sigma) at pH 9.8 or 7.4, as indicated, at room temperature. 195 μl of buffer was added to 5 μl of sample in a 96-well plate (Corning Inc.), and absorbance at 405 nm was evaluated at 5, 10, and 15 min after reaction initiation. *p*-Nitrophenyl phosphate dephosphorylation product nitrophenyl phosphate was compared with serially diluted nitrophenyl phosphate controls of known concentrations, and the rate of nitrophenyl phosphate generation was calculated for each interval (and averaged). AP activity was calculated as units/liter, which is μmol of nitrophenyl phosphate generated/min/liter of plasma (μmol/min/liter). Samples were run in duplicate.

## RESULTS

### 

#### 

##### Blood ATPase and ADPase Activities Are Largely Cell-associated, whereas AMPase Activity Is Largely Plasma-based

**FIGURE 1.**
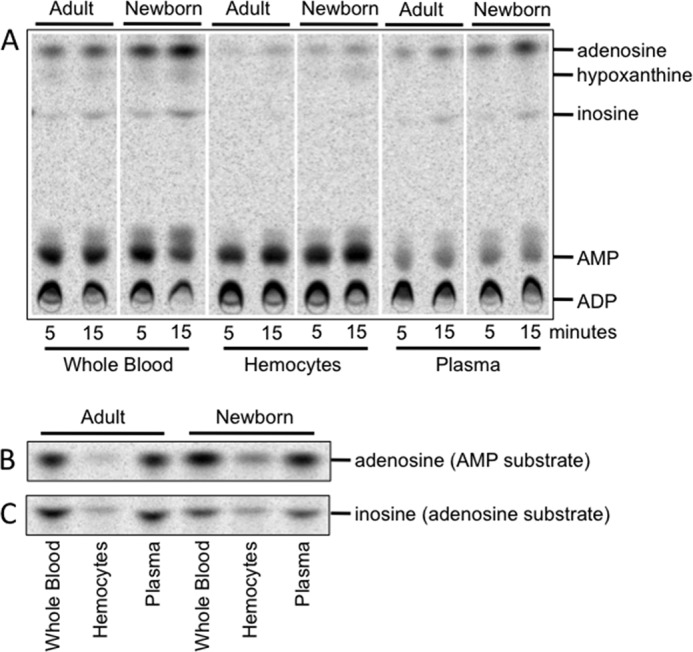
**Cell-associated enzymes dominate newborn and adult blood ADPase activity, whereas soluble enzymes dominate blood AMPase and ADA activities and are differentially expressed in newborns and adults.** Whole blood, hemocytes, or plasma (MFP) were incubated with 50 μm [^14^C]ADP for 5 or 15 min (*A*). MFP was incubated with 50 μm [^14^C]AMP and 20 μm EHNA (*B*) or 50 μm [^14^C]adenosine (*C*) for 15 min prior to measurement of conversion to other purine metabolites by TLC. Shown is one representative of five (*A*) or six (*B* and *C*) independent experiments.

Given the importance of Ado as an endogenous immunoregulatory molecule and the previously discovered difference in Ado concentration between newborn and adult blood plasma (29, 32), we sought to determine if different levels of enzymatic activity in whole blood contribute to the Ado bias in newborn plasma. To this end, radiolabeled ADP ([^14^C]ADP) was added to whole blood, washed hemocytes, or MFP in the presence of 100 μm dipyridamole (to prevent cellular uptake of Ado), and at indicated times, blood-derived supernatants were collected for separation by thin layer chromatography (TLC) as described under “Materials and Methods.” In agreement with previous work employing adult blood (40), these studies indicated that in both neonatal cord and adult peripheral blood, dephosphorylation of ADP to generate AMP was primarily accomplished by cell-associated enzymes, but dephosphorylation of AMP to generate Ado mostly occurred in the soluble phase of blood (Fig. 1*A*). We did not detect significant differences in total AMPase activity in the different preparations of plasma (platelet-rich plasma, platelet-poor plasma, or MFP) but utilized MFP to more completely demonstrate the soluble nature of the enzymatic activity (supplemental Fig. 1). Also, all blood was processed within 2 h of phlebotomy, during which time there was not significant shedding of AP or 5′-NT into the soluble phase of the blood (supplemental Fig. 1).

##### Leukocyte Expression of Purine Ectoenzymes by Flow Cytometry

To determine which cell types in blood expressed the candidate purine-regulating enzymes of interest, we measured CD39 (ENTPD1), CD73 (5′-NT), TNAP, and ADA1 and cell type markers CD3, CD19, CD14, and CD66b by polychromatic flow cytometry as described under “Materials and Methods” (supplemental Fig. 2). In comparison with adults, neonates demonstrated the following differences in subpopulations: (*a*) CD19+ B cells had lower CD73 expression, in agreement with previous work (41), and higher TNAP (supplemental Fig. 2*B*), and (*b*) CD66b+ granulocytes had similar levels of CD39 and no detectable CD73 but significantly more TNAP and less ADA1 (supplemental Fig. 2*C*). Similar levels of CD39 were detected on newborn and adult CD14+ monocytes, whereas CD73, TNAP, and ADA1 were not detected on these cells (supplemental Fig. 2*D*).

##### Robust Neonatal Cord Blood Ado Generation Reflects Greater Cell ATPase/ADPase Activity, Greater Plasma AMPase Activity, and Lower Soluble ADA

**FIGURE 2.**
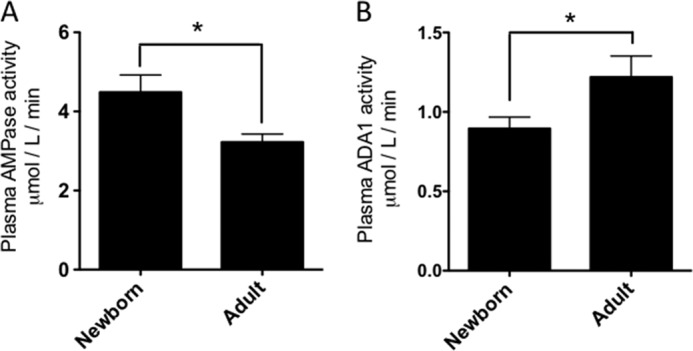
**Neonatal cord blood plasma demonstrates high AMPase and lower relative ADA activities.** *A*, soluble plasma (MFP) AMPase activity was determined by adding 200 μm [^14^C]AMP for 5, 10, and 15 min in the presence of EHNA before reaction termination, and subsequent TLC was quantified by densitometry with the rate calculated based on the change in product between 5 and 10 min and between 10 and 15 min (which were equivalent and were averaged; *n* = 16 each population; Student's *t* test; *, *p* < 0.05). *B*, soluble plasma (MFP) ADA activity was determined by adding 200 μm [^14^C]adenosine for 30 and 60 min prior to reaction termination and TLC separation (*n* = 12 each population; Student's *t* test; *, *p* < 0.05, rate calculated based on the change in product between 30 and 60 min). *Error bars*, S.E.

Newborn blood generated significantly more Ado from ADP than adult blood. ADPase activity in blood is primarily mediated by cellular ENTPD1, and our flow cytometry studies (supplemental Fig. 2) suggested that these activity rates are higher in newborns, due to the well documented elevated leukocyte densities of newborns (42) rather than higher per cell activity. Of note, the robust generation of Ado in newborn blood was also in part due to higher AMPase activity in neonatal blood plasma. To selectively measure AMPase activity, [^14^C]AMP was added to whole blood, washed hemocytes, or MFP in the presence of dipyridamole and 20 μm EHNA (to inhibit deamination of Ado), and the production of Ado was measured by TLC. Although hemocytes did have some AMPase activity, the importance of soluble enzymes for the metabolism of AMP in blood was confirmed (Fig. 1*B*). Similarly, although detectable metabolism of [^14^C]Ado to inosine occurred on hemocytes, substantially greater activity was observed in plasma (Fig. 1*C*; all conditions included dipyridamole). The ADA activity associated with cells and in the plasma was completely EHNA-sensitive, indicating that ADA1 was responsible and not ADA2 (data not shown). AMPase and ADA activity assays, conducted in the absence of dipyridamole, demonstrated that newborn plasma had significantly more AMPase activity on average (Fig. 2*A*), and also significantly less ADA activity (Fig. 2*B*), than adult plasma, both of which could contribute to greater Ado generation in newborn blood. Plasma AMP to adenosine conversion and adenosine deamination were also measured in larger sets of samples with a 50 μm substrate concentration, which may be more relevant in the context of tissue damage *in vivo* (supplemental Fig. 3) (16, 17). Additionally, plasma adenosine deamination was measured with 0.5 μm substrate (supplemental Fig. 4), closer to the basal plasma adenosine levels (32). Preparations of plasma that included microparticles (platelet-poor plasma) and platelets (platelet-rich plasma) did not have AMPase activity (supplemental Fig. 1*A*) or ADA activity^7^ distinguishably different from that of MFP, which was used to evaluate the activity of soluble enzymes that were not associated with particles or platelets. Plasma microparticles do incorporate ENTPD1 (CD39), the activity of which influences endothelial cell activation (43).

##### Soluble AP and CD73 Comprise the Majority of Blood AMPase Activity

**FIGURE 3.**
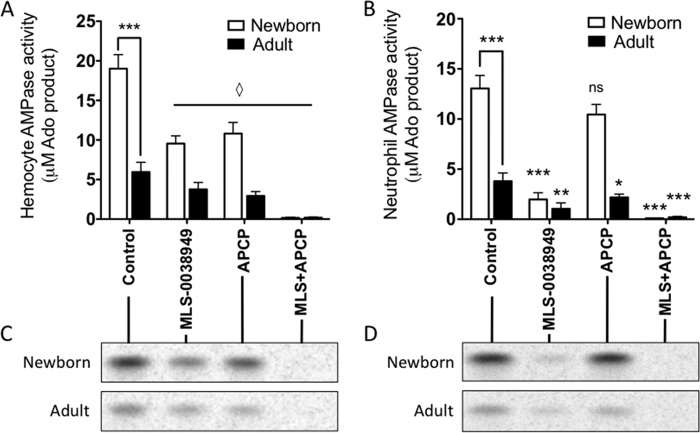
**Newborn neutrophils express relatively high AMPase activity.** Newborn washed hemocytes (*A* and *C*) or isolated peripheral blood neutrophils (4 × 10^6^/ml) (*B* and *D*) were incubated with or without inhibitors of TNAP (MLS0038949; 100 μm) and 5′-NT (APCP; 100 μm) before the addition of 50 μm [^14^C]AMP for 15 min in the presence of EHNA before reaction termination, and subsequent TLC separation was quantified by densitometry. *A*, *n* = 6 for both newborn and adult; *B*, *n* = 5 for newborn and *n* = 6 for adult. *C*, one representative of six independent experiments; *D*, one representative of five independent experiments. *A*, newborn *versus* adult control condition; Student's *t* test; ***, *p* = <0.001; all other conditions compared with appropriate population (newborn or adult) control by analysis of variance with Bonferroni's multiple comparison test (♢) with *p* < 0.01. *B*, newborn *versus* adult Student's *t* test; *, *p* < 0.05; **, *p* < 0.01; ***, *p* < 0.001, all other conditions compared with population control by analysis of variance with Bonferroni's multiple comparison test, with *p* < 0.01 unless indicated as not significant (*ns*). *Error bars*, S.E.

To assess the relative contribution of soluble and cell-associated enzymes to blood AMPase activity, [^14^C]AMP (50 μm) was added to either cell (*i.e.* hemocyte) or plasma blood fractions, with or without the addition of inhibitors of 5′-NT (APCP 100 μm) or TNAP (MLS-0038949 (44) 100 μm), and conversion to Ado was assessed. Of note, in both newborn and adult blood studied *ex vivo*, only a minority of blood AMPase activity was cell-associated. Upon incubation with 100 μm MLS-0038949, 100 μm APCP, or both and 50 μm [^14^C]AMP, newborn hemocytes demonstrated a significantly higher conversion of AMP to adenosine than adult hemocytes (Fig. 3). Experiments were performed at volumetric equivalence (*i.e.* not at equivalent cell numbers), and differences in cell density, known to be higher in newborn blood (42), probably contribute to observed differences in purine metabolism. Surprisingly, AP was responsible for a slight majority of newborn hemocyte-associated AMPase activity, with 5′-NT responsible for the remainder (Fig. 3, *A* and *C*). In contrast, adult hemocytes demonstrated a reverse pattern, with 5′-NT responsible for a slight majority of total activity. In neutrophils isolated from peripheral venous blood, AMP conversion to adenosine was largely due to AP activity, with modest 5′-NT activity (Fig. 3, *B* and *D*). Neutrophil AP-mediated ecto-AMPase activity was significantly higher on newborn cells than on adult cells. RBCs had very low levels of AMPase activity (data not shown) but represent the considerable majority of hemocyte ecto-ADA activity. Newborn and adult RBC ecto-ADA1 activity rates per cell, as assessed by measuring conversion of [^14^C]Ado to inosine as described under “Materials and Methods,” were not significantly different (data not shown).

##### The Majority of Blood AMPase Activity Is Soluble

**FIGURE 4.**
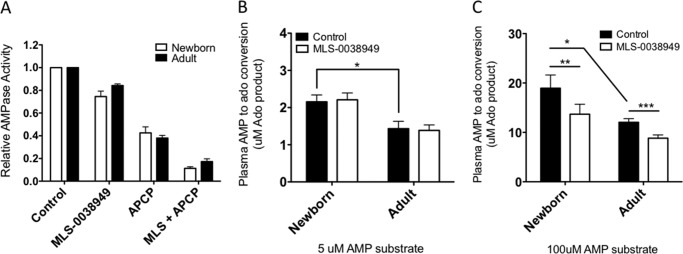
**TNAP and 5′-NT are key contributors to soluble plasma AMPase activity.** The contributions of TNAP and 5′-NT were determined by the addition of a 100 μm concentration of the selective inhibitor MLS0038949 or APCP, respectively. 50 μm [^14^C]AMP was added in the presence of EHNA, and after 5 min, the reaction was terminated prior to TLC separation and quantified by densitometry (*A*; *n* = 11). TNAP did not influence total AMPase activity in plasma at low concentrations of substrate (*B*; 5 μm [^14^C]AMP, 1 min, *n* = 8) but did significantly contribute at high concentrations (*C*; 100 μm [^14^C]AMP; 5 min; *n* = 8; Student's *t* tests; *, *p* < 0.05; **, *p* < 0.01; ***, *p* < 0.001). *Error bars*, S.E.

To determine which enzymes were responsible for the AMPase activity in plasma, the conversion of [^14^C]AMP (added at 50 μm) to Ado in plasma (MFP) was assessed with or without the addition of inhibitors of 5′-NT (APCP; 100 μm) or TNAP (MLS-0038949 (44); 100 μm) as described under “Materials and Methods.” Although 5′-NT was primarily responsible for soluble AMPase activity, TNAP also significantly contributed (Fig. 4*A*). Because previous work (40) utilizing 1 μm AMP substrate had ruled out a role for AP in plasma AMPase activity, we evaluated whether AP contributed to AMPase activity only at higher substrate concentrations. Although at a 5 μm AMP concentration (*i.e.* basal AMP levels), AP did not substantially contribute (Fig. 4*B*), at a 100 μm (*i.e.* pathophysiologic) AMP concentration, AP significantly contributed to plasma AMPase activity (Fig. 4*C*). In fact, the plasma AMPase rate, calculated with a substrate concentration of 200 μm, demonstrates a significant positive relationship with plasma alkaline phosphatase activity (supplemental Fig. 5). These observations suggest that plasma AP, known to be higher at birth (45), may play a substantial role in blood Ado generation when extracellular levels of purine nucleotides are substantially elevated during tissue damage or in a hypoxic environment. Thus, soluble AP appears to be critically involved in blood Ado production at elevated levels of extracellular purine precursors and to a greater extent in newborn than in adult blood; reduction in AMPase activity with TNAP inhibitor (MLS-0038949) for newborn plasma was 0.70 ± 0.56 μmol/liter/min compared with 0.31 ± 0.13 μmol/liter/min (*p* < 0.05; Student's *t* test; *n* = 11 each population; shown as mean ± S.D., calculated from the Fig. 4*A* data set). We characterized the expression of AP isoforms at birth and found that neonatal plasma contains greater levels of TNAP (as expected (46)) and a distinct TNAP isoform profile compared with adult plasma (supplemental Fig. 6). The majority of total AP at birth was the bone isoform of TNAP, although relative to adult plasma, neonatal plasma also contained high concentrations of the liver type 2 isoform of TNAP and lower liver TNAP isoform 1. We also confirmed that higher alkaline phosphatase activity in neonatal as compared with adult plasma was also noted when assayed at pH 7.4, further indicating the physiologic relevance of these findings.^7^

##### Age-dependent Increase in ADA during Infancy

**FIGURE 5.**
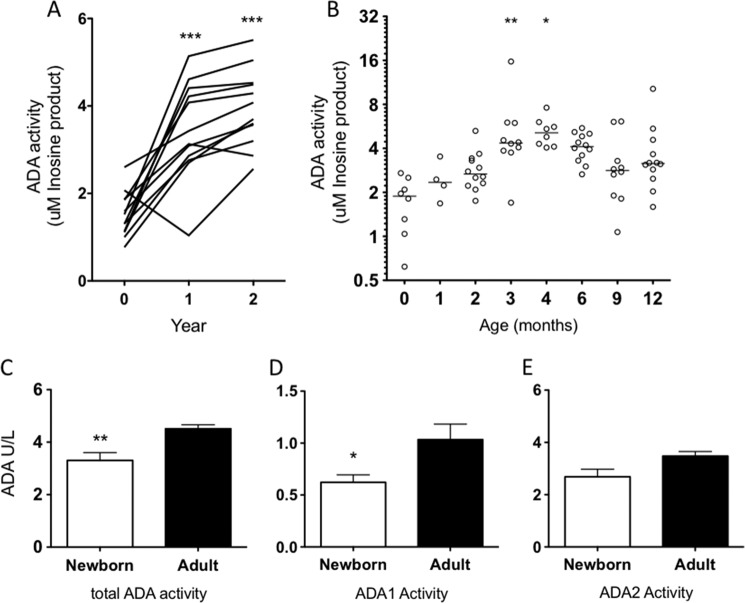
**Soluble plasma ADA levels increase significantly during the first year of life.** *A*, soluble ADA activity was determined by the addition of 50 μm [^14^C]adenosine for 5 min to microparticle-free plasma collected at 0, 1, and 2 years of age from 12 subjects (paired Student's *t* tests; 0 *versus* 1, *p* < 0.001; 0 *versus* 2, *p* < 0.0001; 1 *versus* 2, *p* < 0.01). *B*, a cohort of platelet-rich plasma samples from a previous study, including samples from cord blood and 1, 2, 3, 4, 6, 9, and 12 months of age (1 sample/subject), were processed to MFP and incubated with 50 μm [^14^C]adenosine for 15 min (analysis of variance with Bonferroni's multiple comparison test; 0 *versus* 3 months, *p* < 0.01; 0 *versus* 4 months, *p* < 0.05). Total ADA (*C*), ADA1 (*D*), and ADA2 (*E*) activity were evaluated by a chromogenic assay as described under “Materials and Methods.” Total ADA (*n* = 11 newborn, *n* = 14 adult; Student's *t* test; **, *p* < 0.01) (*C*) and ADA1 activity (*n* = 11 newborn; *n* = 14 adult; Student's *t* test; *, *p* < 0.05) (*D*) were significantly lower in newborn samples. *Error bars*, S.E.

We next characterized the ontogeny of soluble ADA1 and 5′-NT in two cohorts of infant plasma samples: (*a*) plasma collected in a cross-sectional study at birth and at 1, 2, 3, 4, 6, 9, and 12 months and (*b*) plasma collected in a separate longitudinal study at birth and at 1 and 2 years of age. ADA activity was assayed as described previously with [^14^C]Ado substrate. Of note, the plasma adenosine deamination was significantly higher at 1 and 2 years of age than at birth (Fig. 5*A*). In the cross-sectional study of West African (Gambian) infants, plasma adenosine deamination was significantly increased by 3 months of age as compared with birth (cord; Fig. 5*B*). A chromogenic ADA assay utilizing high concentrations of Ado substrate revealed that total ADA and ADA1 activities were significantly lower in newborn plasma, in agreement with our assay at more physiological concentrations of substrate (Fig. 5, *C–E*). Activity in neonatal plasma of ADA2, a protein with weak deaminase activity (47) that probably functions rather as a growth factor (48), was not significantly different between newborn and adult plasma. No significant changes were observed in soluble 5′-NT activity in plasma during the first 2 years of life in the above described infant cohorts (data not shown).

##### Inhibition of Blood 5′-NT Activity Enhances whereas the Addition of AMP Inhibits TLR-mediated TNF-α Production in Blood

To assess the potential functional relevance of robust Ado-generating activity in human newborn cord blood, we tested the effect of selective inhibition of 5′-NT. To this end, human newborn cord and adult peripheral blood were incubated with vehicle control or with the 5′-NT-selective inhibitor APCP. Because our prior studies suggested that Ado may particularly affect polarization of TLR2-mediated cytokine production (32), we assessed the impact of 5′-NT inhibition on cytokine induction by *S. epidermidis*, a Gram-positive pathogen that causes neonatal bacteremia and signals via TLR2 (49). Consistent with a role for Ado in inhibiting neonatal TNF-α induction, the addition of APCP significantly and selectively enhanced *S. epidermidis*-induced production of the proinflammatory/Th1-polarizing cytokine TNF-α in newborn whole blood (supplemental Fig. 7). In contrast, *S. epidermidis*-induced production of IL-1β and of the Th17-polarizing cytokines IL-6 and IL-23 and of the anti-inflammatory cytokine IL-10 was not affected by the addition of APCP, indicating cytokine-specific Ado effects on neonatal leukocytes. It should be noted that *S. epidermidis* expresses a 5′-NT homologue (50), AdsA, which is also inhibited by APCP and would have contributed to AMPase activity in this assay. A modest enhancement of newborn TNF-α in response to LPS (TLR4) in the presence of APCP did not reach significance, and the other cytokines tested were unaffected (data not shown). TNF-α in control whole blood samples was low but detectable and was not significantly different between newborn and adult samples (data not shown).

**FIGURE 6.**
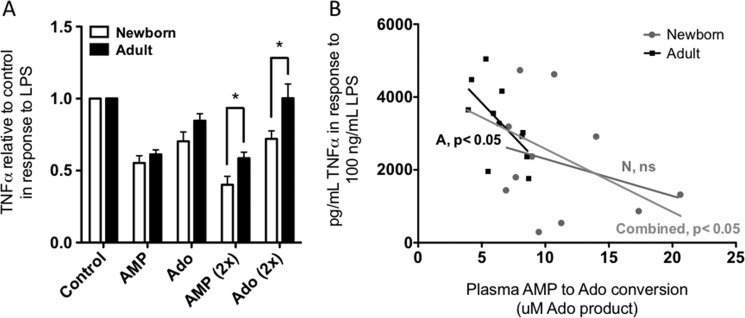
Extracellular AMP metabolism leads to diminished LPS-induced TNF-α production in newborn and adult blood (*A*). Newborn cord or adult peripheral blood was diluted 1:1 (final) with RPMI and treated with LPS (100 ng/ml) or mock-treated with RPMI and treated with 100 μm AMP or Ado once (at start) or twice (second addition at 2 h), as indicated, and all wells were mixed gently at the 2 h time point. After a total of 4 h of incubation (37 °C, 5% CO_2_, 96-well round bottom plates), supernatants were evaluated by ELISA for TNF-α (*n* = 13 newborn, *n* = 15 adult; Student's *t* test; *, *p* < 0.05). Whole blood samples with higher plasma AMPase activity produce less TNF-α in response to LPS (*B*). Plasma AMP dephosphorylation to adenosine was determined as in Fig. 3; *n* = 11 for each population; statistical analysis by linear regression, significantly non-zero slope for adult and combined populations; *, *p* < 0.05; newborn population not significant. *Error bars*, S.E.

Additionally, we have evaluated the ability of AMP or Ado to modulate the production of proinflammatory cytokine (TNF-α) in a 96-well plate format whole blood assay. Newborn cord or adult peripheral blood was diluted with RPMI and treated with LPS (TLR4 agonist; 100 ng/ml) or mock-treated with RPMI in the presence or absence of 100 μm AMP or Ado. Additional wells were treated again with 100 μm AMP or Ado 2 h after the initial stimulation (other wells received an addition of RPMI at this time point, and all wells were mixed gently). After a total of 4 h of incubation, cells were pelleted by centrifugation at 500 × *g* for 5 min, and supernatants were evaluated by ELISA for TNF-α (Fig. 6). Because no receptor has been described for AMP, the effect of AMP is presumed to be mediated by Ado following dephosphorylation of AMP. AMP and Ado addition diminished TNF-α production. In samples treated twice, AMP and Ado were significantly more inhibitory in newborn cord than in adult peripheral blood. The increased sensitivity in the newborn population to AMP may be due to elevated AMPase activity or greater sensitivity to the metabolic product Ado, whereas increased sensitivity to Ado may also be related to a deficiency in the newborn samples of ADA, resulting in slower clearance of Ado. We evaluated the plasma AMPase activity from the same blood samples used in Fig. 6*A* to determine if there was a correlation between plasma AMPase activity and TNF-α production. There was a significant, negative relationship between plasma AMPase activity and whole blood TNF-α production; samples with higher AMPase activity produced less TNF-α in response to LPS (Fig. 6*B*).

## DISCUSSION

Our study has characterized Ado-generating and metabolizing activity in human neonatal blood. eATP and eAdo signaling impacts and regulates a wide variety of cellular functions, and thus the regulation of these molecules in the extracellular space is critical. Because eAdo is elevated in newborn blood, which potentially influences differential immunological function in this population (28), we assessed whether newborn eAdo regulation in blood differed from that in adults and identified the enzymes involved. We found that ATPase and ADPase activity in whole blood is primarily mediated by cell-associated ENTPD1 (CD39) (51), and although such hemocyte activity was higher by volume in newborn blood (Fig. 1*A*), this may reflect greater numbers of leukocytes at birth (52). Thus, eATP and extracellular ADP dephosphorylation appears to be higher in newborn blood due to elevated numbers of leukocytes, which express similar levels of ENTPD1 per cell compared with adult cells. AP significantly contributes to cell-associated AMPase activity and, at elevated levels of AMP (≥50 μm), comprises the majority of cellular AMPase activity in blood at birth (Fig. 4, *B* and *C*). Although we found modest levels of AP expression on subsets of circulating lymphocytes by flow cytometry (supplemental Fig. 1*A*), CD66b + granulocytes (primarily neutrophils) express abundant AP but low CD73 at birth (for expression, see supplemental Fig. 1*C*; for activity, see Fig. 3*B*). Because purinergic signaling and ectopurine metabolism are critical for several aspects of neutrophil function (9), including chemotaxis (53), future work will focus on the functional implications of greater granulocyte-associated AP expression at birth.

**FIGURE 7.**
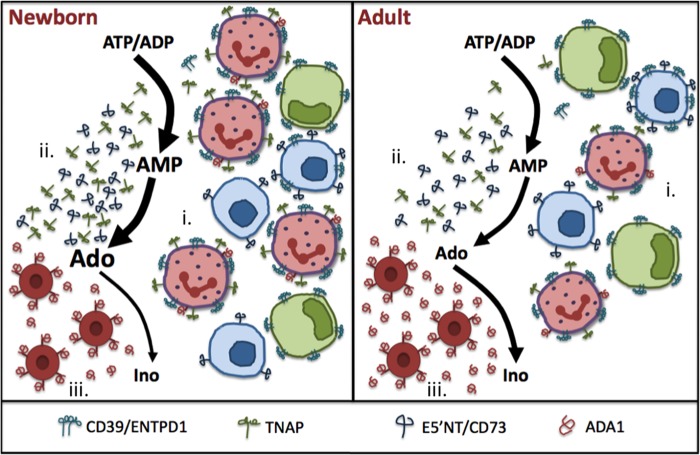
**Distinct purine metabolism at birth results in higher ambient adenosine concentrations.** This figure presents a model that synthesizes both published and novel information from this current study highlighting age-specific differences in sequential cell- and plasma-based generation of Ado from ATP and of deamination of Ado to inosine (*Ino*). *A*, newborn blood contains a high density of CD39/ENTPD1-expressing leukocytes, contributing to high ATPase and ADPase activity (*i*); high plasma concentration of 5′-NT and AP that drive conversion of AMP to Ado (*ii*); and relative deficiency of ADA, resulting in a net increase in ambient adenosine concentrations and capacity to generate adenosine following release of purine substrates (*iii*). *B*, in marked contrast, adult blood features lower density of CD39/ENTPD1-expressing leukocytes (*i*), lower plasma 5′-NT and AP activity (*ii*), and relatively greater ADA activity, resulting in lower basal adenosine concentrations (*iii*).

In marked contrast to the dephosphorylation of ATP and ADP in blood that is largely cell-based, extracellular dephosphorylation of AMP in newborn cord and adult peripheral blood is primarily accomplished by soluble plasma factors, specifically soluble 5′-NT and APs (see model in Fig. 7). Of note, 5′-NT is also expressed on endothelial cells (11), and *in vivo*, this may significantly impact blood AMPase activity.

There are four human AP genes: *ALPL* (tissue-nonspecific AP, high levels in bone, kidney, and liver but widely expressed in other tissues as well), *ALPP* (placental AP), *ALPPL2* (germ cell AP), and *ALPI* (intestinal AP) (45). We found that the majority of total AP at birth was the bone isoform of the *ALPL* gene product (TNAP), although relative to adult plasma, neonatal plasma also contained relatively high concentrations of liver type 2 isoform TNAP and lower liver TNAP isoform 1 (supplemental Fig. 6). Serum AP levels, predominantly the bone isoform of TNAP early in life, are elevated during infancy and childhood compared with adulthood (54). In agreement with our results (Fig. 3*B*), TNAP did not measurably contribute to AMP hydrolysis in blood at 1 μm AMP substrate (40). However, at elevated levels of the purine substrate AMP (>50 μm), AP significantly contributed to purine metabolism (Fig. 3*C* and supplemental Fig. 5). There appears to be a wide range of (patho)physiologically relevant purine concentrations *in vivo*. Low concentrations of eATP (0.01–0.1 μm) are released from resting cells, and transient moderate increases in eATP (to <5 μm) mediate critical cell signaling functions (17). Larger releases of eATP (*e.g.* in the context of cellular damage) constitute a danger signal, reaching ∼10–20 μm in rat and human blood following vascular injury (55) and >100 μm in murine tumor interstitia (56). *In vitro* EC_50_ values for the P2X and P2Y eATP receptors have been estimated to be as low as 0.05 μm for P2X_1_ and as high as 780 μm for P2X_7_ (9), which plays a critical role in inflammatory responses to intracellular pathogens (57, 58). eAdo is generated primarily via metabolism of eAMP from extracellular ADP and eATP released from platelets and cells (9, 17). EC_50_ values for the adenosine receptors have been estimated to be as low as 0.18 μm (A_1_) and as high as 64.1 μm (A_2B_) (9). eAdo concentrations in the murine central nervous system are typically in the 0.01–0.1 μm range but upon systemic insult increase into the >1 μm range (59), having been measured as high as 40 μm (60). Working within the limits of detection of the thin layer chromatography assay, we have herein studied enzymatic regulation of extracellular purines in blood and blood plasma *ex vivo* utilizing substrate concentrations at 0.5 μm eAdo, close to basal plasma eAdo concentrations (∼0.15 and 0.05 μm eAdo in newborns and adults, respectively (32)). For the purposes of measuring enzymatic rates at elevated and saturating concentrations, we have also characterized these plasma enzymes at 50 and 200 μm eAdo and eAMP, which are relatively high purine substrate concentrations that might be achieved locally during pathophysiologic states of injury and inflammatory tissue damage. Overall, our results indicate that neonatal purine-metabolizing enzymatic activity is significantly and consistently different from that of adults across a range of substrate concentrations, such that neonatal plasma will generate more eAdo and metabolize less of that eAdo to inosine, resulting in the higher ambient eAdo concentrations. Our results are probably valid in that (*a*) age-dependent maturation of ADA expression was observed across multiple and diverse newborn and infant cohorts in Boston, The Gambia (West Africa), and British Columbia, and (*b*) they are consistent with the higher newborn than adult plasma eAdo concentrations measured *ex vivo* (32).

Overall, enzymatic generation of eAdo is higher in newborn blood than in adult blood, and enzymatic deamination of eAdo is lower, favoring elevated levels of eAdo in this population (see model in Fig. 7). Of note, we also demonstrate that higher soluble AMPase activity correlates with lower LPS-induced proinflammatory TNF-α production in blood stimulated with LPS (Fig. 6*B*). Thus, the endogenous purine enzyme profile may critically regulate innate immune responses. Moreover, consistent with our prior studies (32), neonatal leukocytes appeared to be more sensitive to the inhibitory effects of Ado in that addition of AMP resulted in more profound inhibition of LPS-induced TNF production in newborn than in adult blood. Our demonstration that inhibition of 5′-NT activity with APCP enhances TNF production induced by *S. epidermidis*, a bacterium that activates human leukocyte cytokine production via TLR2 (49), also suggests that such eAdo generation may be functionally relevant, contributing to suppression of proinflammatory/Th1-polarizing cytokine while not influencing Th17-polarizing cytokines, such as IL-6, and anti-inflammatory cytokines, such as IL-10 (supplemental Fig. 7). Such patterns of low Th1 but preserved Th2/Th17/anti-inflammatory fetal/neonatal cytokine production have been observed *in vitro* and *in vivo* (35, 61, 62). This cytokine skew may serve to limit fetal-maternal alloimmune reactions and excessive inflammation upon initial colonization in early life but may also render the newborn more susceptible to infections with intracellular pathogens and impair vaccine responses.

The immunosuppressive activity of Ado is abrogated by ADA-mediated deamination of Ado to generate inosine. ADA1 (ADA) is primarily responsible for the deamination of Ado at physiological levels of substrate (19), whereas both ADA1 and ADA2 (*CECR1*) have co-stimulatory and growth factor activities. Extracellular ADA is found either soluble in fluids or cell-associated via binding to CD26 (26), or Ado receptors (27). ADA1 expression is not well characterized in newborns unless it is entirely absent, as in ADA deficiency causing severe combined immunodeficiency (63). We have discovered a physiologic deficiency of soluble plasma ADA at birth and characterized the age-dependent maturation of plasma ADA expression across two international cohorts, revealing that levels of this key enzyme gradually rise during the first year of life. Whereas the uncommon genetic deficiency of ADA severely impairs multiple immune responses in effected individuals, our discovery of a relative physiologic ADA deficiency that is apparently normal at birth and early infancy suggests a potential mechanism that may contribute to the general susceptibility of healthy newborns to bacterial, mycobacterial, and viral infections (64). Of note, a similar profile (elevated plasma AP (65), 5′-NT (66), and eAdo (66) and lower total ADA (but not lower ADA1) (67)) is found during pregnancy, another functionally distinct immunologic state associated with impaired Th1-polarizing cytokine responses and enhanced susceptibility to intracellular pathogens (68, 69).

Our results may also have translational implications. First, a great deal of preclinical biomedical research directed at development of immunomodulatory agents, such as immune response modifiers, adjuvants, and vaccines, employs *in vitro* assays of leukocytes. To the extent that eAdo exerts relevant immunomodulatory effects, such efforts to model age-specific immune responses *in vitro* should take into account that plasma demonstrates age-specific Ado-generating activity. Accordingly, rather than culturing the leukocytes to be compared in artificial media or heat-treated pooled adult sera, as is often done, such assays may be optimally conducted by employing age-specific plasma matched to the age of the individuals from which the leukocytes are isolated. From a therapeutic standpoint, multiple purine analogues, such as adenosine, caffeine, and imiquimod, are used clinically, and several others are in biopharmaceutical development. Imidazoquinolines, such as the FDA-approved topical antiviral agent imiquimod, are small synthetic purine analogues that activate TLR7 and/or TLR8, which also interact with the Ado system (70–72). Certain imidazoquinolines that are relatively more refractory to the inhibitory effects of Ado, such as the TLR7/8 agonist R-848 (resiquimod), are particularly effective in activating human neonatal monocytes and monocyte-derived dendritic cells, suggesting potential as neonatal immune response modifiers and/or vaccine adjuvants (73). To the extent that enhancement of neonatal immune responses may be beneficial, as has been demonstrated in neonatal murine studies (74), agents that are relatively refractory to Ado inhibition or that inhibit AP and/or 5′-NT (thereby reducing generation of Ado) may enhance neonatal host defense (75).

## Supplementary Material

Supplemental Data

## Footnotes

7 M. Pettengill, S. Robson, M. Tresenriter, J. L. Millán, A. Usheva, T. Bingham, M. Belderbos, I. Bergelson, S. Burl, B. Kampmann, L. Gelinas, T. Kollmann, L. Bont, and O. Levy, unpublished observations.

## Footnotes

6 The abbreviations used are:

eATP: extracellular ATP
Ado: adenosine
eAdo: extracellular Ado
ENTPD: ectonucleoside triphosphate diphosphohydrolase
AP: alkaline phosphatase
5′-NT: ecto-5′-nucleotidase
TLR: Toll-like receptor
TNAP: tissue-nonspecific alkaline phosphatase
APCP: adenosine 5′-(α,β-methylene)-diphosphate
HBSS: Hanks' buffered salt solution
EHNA: *erythro*-9-(2-hydroxy-3-nonyl)adenine hydrochloride
dipyridamole: 2,6-bis(diethanolamino)-4,8-dipiperidinopyrimido[5,4-*d*]pyrimidine
MFP: microparticle-free plasma
RBC: red blood cell
ADA: adenosine deaminase
TLC: thin layer chromatography.
