# Supplementary material for: Soluble Ecto-5′-nucleotidase (5′-NT), Alkaline Phosphatase, and Adenosine Deaminase (ADA1) Activities in Neonatal Blood Favor Elevated Extracellular Adenosine
Source: J Biol Chem. 2013 Jul 29;288(38):27315–26. doi: 10.1074/jbc.M113.484212 (PMC3779727; doi:10.1074/jbc.M113.484212)
Supplement: Supplemental Data [file supp_288_38_27315__index.html]

Soluble ecto-5′-nucleotidase (5′NT), alkaline phosphatase, and adenosine deaminase (ADA1) activities in neonatal blood favor elevated extracellular adenosine — Soluble Ecto-5′-nucleotidase (5′-NT), Alkaline Phosphatase, and Adenosine Deaminase (ADA1) Activities in Neonatal Blood Favor Elevated Extracellular Adenosine — Anti-inflammatory Purine Metabolism Profile in Newborn Blood — Supplemental Data 

# Soluble Ecto-5′-nucleotidase (5′-NT), Alkaline Phosphatase, and Adenosine Deaminase (ADA1) Activities in Neonatal Blood Favor Elevated Extracellular Adenosine

## Supplemental Data

**Files in this Data Supplement:**

- Supplemental Figures (.pdf, 762 KB) - Supplemental Figures 1-7 and corresponding figure legends
