# Supplementary material for: Soluble Ecto-5′-nucleotidase (5′-NT), Alkaline Phosphatase, and Adenosine Deaminase (ADA1) Activities in Neonatal Blood Favor Elevated Extracellular Adenosine
Source: J Biol Chem. 2013 Jul 29;288(38):27315–26. doi: 10.1074/jbc.M113.484212 (PMC3779727; doi:10.1074/jbc.M113.484212)
Supplement: Supplemental Data [file supp_M113.484212_jbc.M113.484212-1.pdf]

Supplementary Figure Legends:

Supplementary Figure 1: Evaluation of plasma preparation AMPase activity and blood enzyme shedding. Adult peripheral blood or newborn cord blood was collected and processed for plasma as described in methods, with initiation of plasma separation 15 minutes or 2 hours after phlebotomy, as indicated. Total AMPase activity determined as indicated for Figure 2A. Alkaline phosphatase activity was determined by p-NPP hydrolysis at both pH 9.8 and pH 7.4, as described in methods. There were no significant differences between plasma preparations, and no significant difference in soluble AMPase activity with extended time before preparation was initiated (at 2 hours). N=2 each population (newborn and adult), combined analysis.

Supplementary Figure 2: Newborn cord blood leukocytes express distinct levels of purine metabolizing enzymes as compared to adult peripheral blood leukocytes. Whole newborn cord or adult peripheral blood was stained with cell lineage marker antibodies for lymphocytes (**A**, CD3+ T cells, **B**, CD19+ B cells), granulocytes (**C**, CD66b+), and monocytes (**D**, CD14+), as well as enzyme targeted antibodies for CD39 (ENTPD1), CD73 (5'NT), TNAP, and ADA1. Cell type gating was also based on light scattering properties. Only CD66b+ cells were greater than 5% positive for ADA1. N=7 for both adult and newborn, except N=4 for ADA1 staining (both populations). Student's t-tests, * $p < 0.05$, ** $p < 0.01$, *** $p < 0.001$.

Supplementary Figure 3: Neonatal cord blood plasma demonstrates high AMPase and lower relative adenosine deaminase (ADA) activities. (A) Soluble plasma (MFP) AMPase activity was determined by adding 50 μM AMP (C^{14}) for 1 minute in the presence of EHNA before reaction termination, and subsequent TLC was quantified by densitometry (N=41 each population, Student's t-test, *** p = <0.001). (B) Soluble plasma (MFP) ADA activity was determined by adding 50 μM adenosine (C^{14}) for 15 minutes prior to reaction termination and TLC separation (N=31 each population, student's t-test ***$p < 0.001$).

Supplementary Figure 4: Neonatal cord blood plasma demonstrates lower adenosine deaminase (ADA) activity than adult blood plasma at low levels of adenosine substrate. Soluble plasma (MFP) ADA activity was determined by adding 0.5 μM adenosine (C^{14}) for 30 minutes prior to reaction termination and TLC separation (n=24 each population, student's t-test ***$p < 0.001$).

Supplementary Figure 5: Positive correlation between plasma alkaline phosphatase (AP) activity and plasma total AMPase rate. Plasma AMPase rate determined as in Figure 2A with 200 μM AMP (C^{14}) , and AP activity evaluated as described in methods at pH 9.8. N = 16 for each population, statistical analysis by linear regression, significantly non-zero slope for adult ** $p<0.01$, and combined populations, *** $p<0.001$, newborn population alone not significant.

Supplementary Figure 6: Newborns have elevated levels of soluble AP in blood, and display a unique TNAP isoform profile compared to adults. Serum samples were assayed for alkaline phosphatase isoform content by electrophoresis as described in Methods. Results were expressed as AP U/L for bone, liver type1, and liver type 2 AP. Newborns had significantly greater bone, liver 2 and total AP than adults. Conversely, adults had greater liver 1 AP. Intestinal AP was detected in 1 adult sample (10 U/L) while no placental or germ-cell AP was detected. Liver type 1 AP was not detected in 3 out of 6 newborn samples. N=6, Student's t-tests, adult vs. newborn; Bone, *** $p < 0.001$, Liver 1, ** $p<0.01$, Liver 2, *** $p < 0.001$, Total AP, *** $p < 0.001$.

Supplementary Figure 7: Inhibition of 5'NT selectively enhances *Staphylococcus epidermidis*-induced TNF-α production in newborn whole blood. Whole cord blood was incubated with APCP (100 μM) or buffer control before stimulation with 10^7 SE per mL or vehicle control for 4

hours during end-over-end rotation at 37°C. Supernatants were collected following centrifugation and TNF-α, IL-6, IL-1β, IL-23, and IL-10 were measured by ELISA. Displayed as mean plus SEM for ratio of cytokine detected with/without APCP incubation. IL-10 was below the limits of detection for the majority of adult samples. N = 5-8 (N=7 for TNF-α), ** $p < 0.01$.

FIG S1

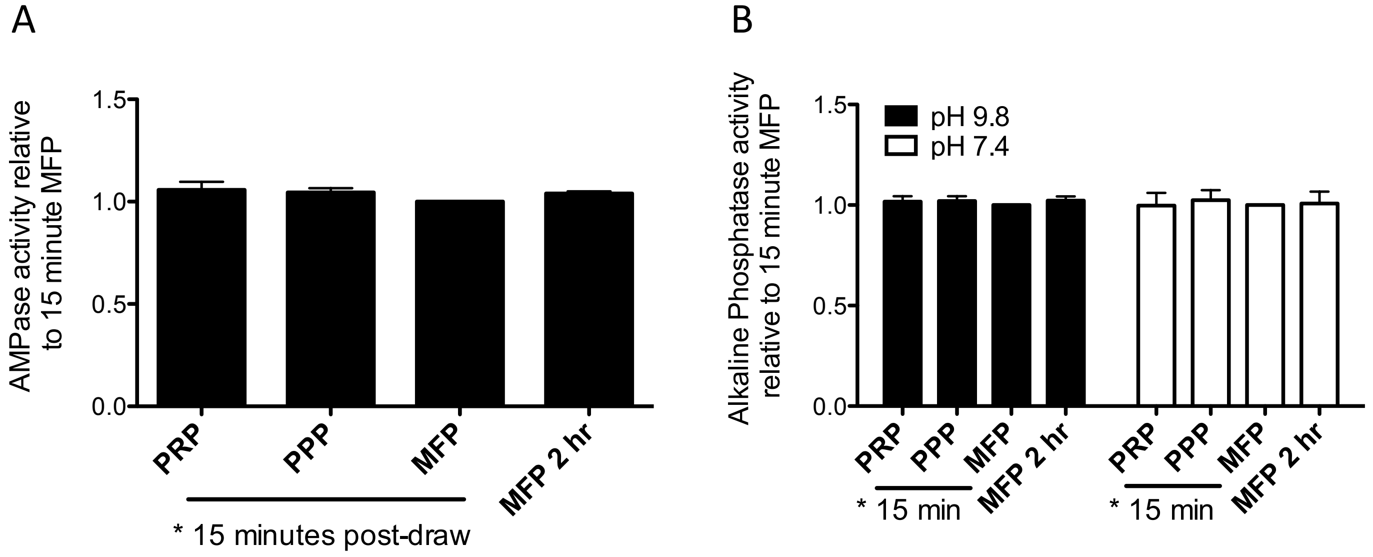
A
AMPase activity relative to 15 minute MFP
1.5
1.0
0.5
0.0
PRP
PPP
MFP
MFP 2 hr
* 15 minutes post-draw
B
Alkaline Phosphatase activity relative to 15 minute MFP
pH 9.8
pH 7.4
1.5
1.0
0.5
0.0
PRP
PPP
MFP
MFP 2 hr
* 15 min
PRP
PPP
MFP
MFP 2 hr
* 15 min

FIG S2

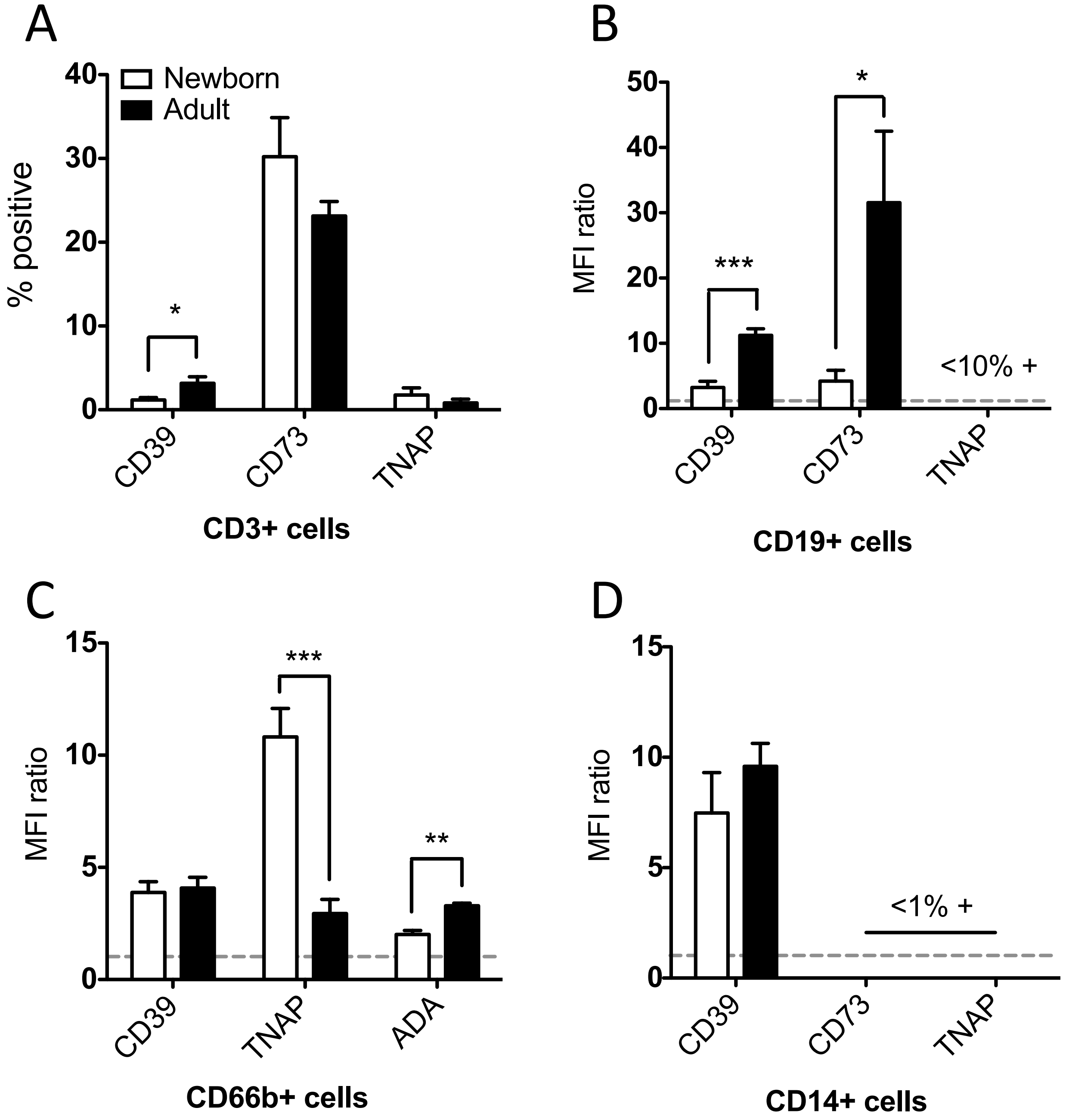
A
40
30
20
10
0
% positive
Newborn
Adult
*
CD39
CD73
TNAP
CD3+ cells
B
50
40
30
20
10
0
MFI ratio
***
*
<10% +
CD39
CD73
TNAP
CD19+ cells
C
15
10
5
0
MFI ratio
***
**
CD39
TNAP
ADA
CD66b+ cells
D
15
10
5
0
MFI ratio
<1% +
CD39
CD73
TNAP
CD14+ cells

FIG S3

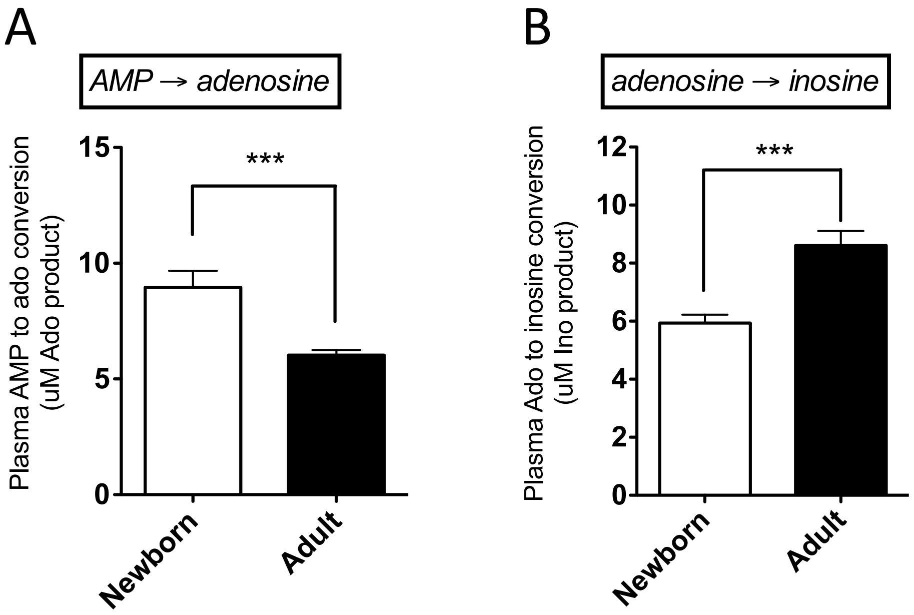
A
AMP → adenosine
Plasma AMP to ado conversion (uM Ado product)
15
10
5
0
***
Newborn
Adult
B
adenosine → inosine
Plasma Ado to inosine conversion (uM Ino product)
12
10
8
6
4
2
0
***
Newborn
Adult

FIG S4

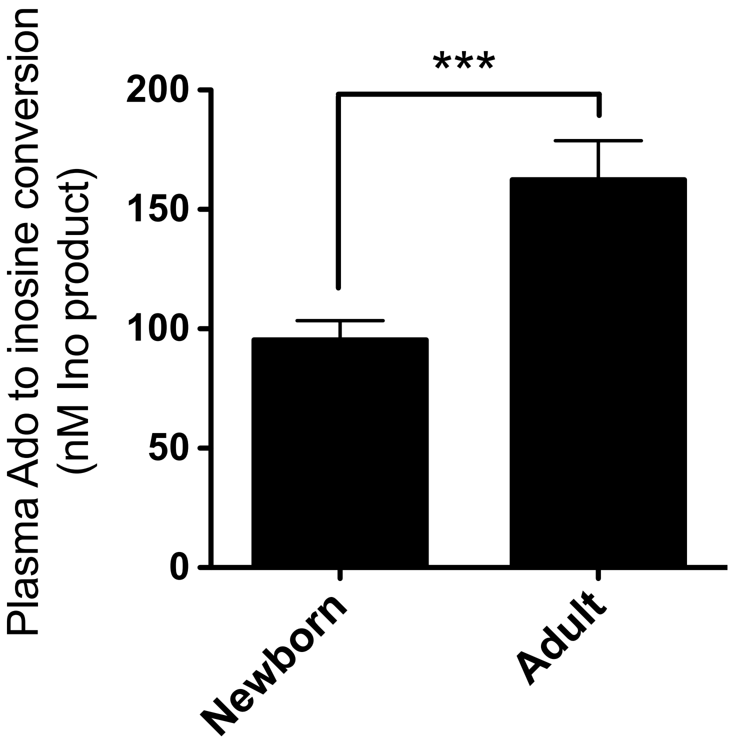
Plasma Ado to inosine conversion
(nM Ino product)
200
150
100
50
0
***
Newborn
Adult

FIG S5

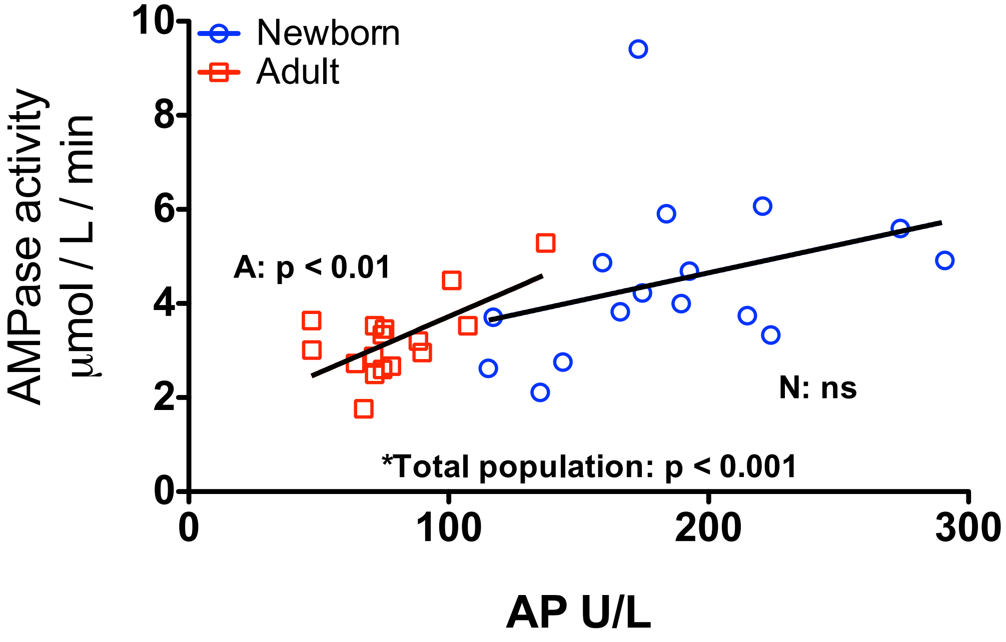
Newborn
Adult
A: p < 0.01
N: ns
*Total population: p < 0.001
AMPase activity
μmol / L / min
AP U/L
0
2
4
6
8
10
0
100
200
300

FIG S6

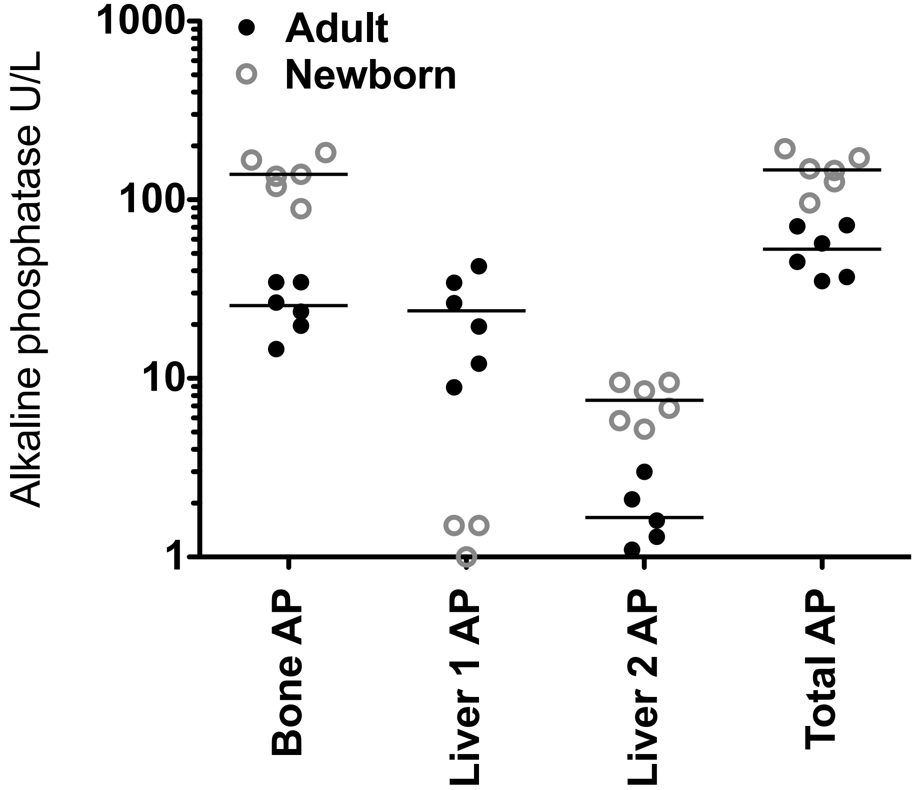
1000
100
10
1
Alkaline phosphatase U/L
Adult
Newborn
Bone AP
Liver 1 AP
Liver 2 AP
Total AP

FIG S7

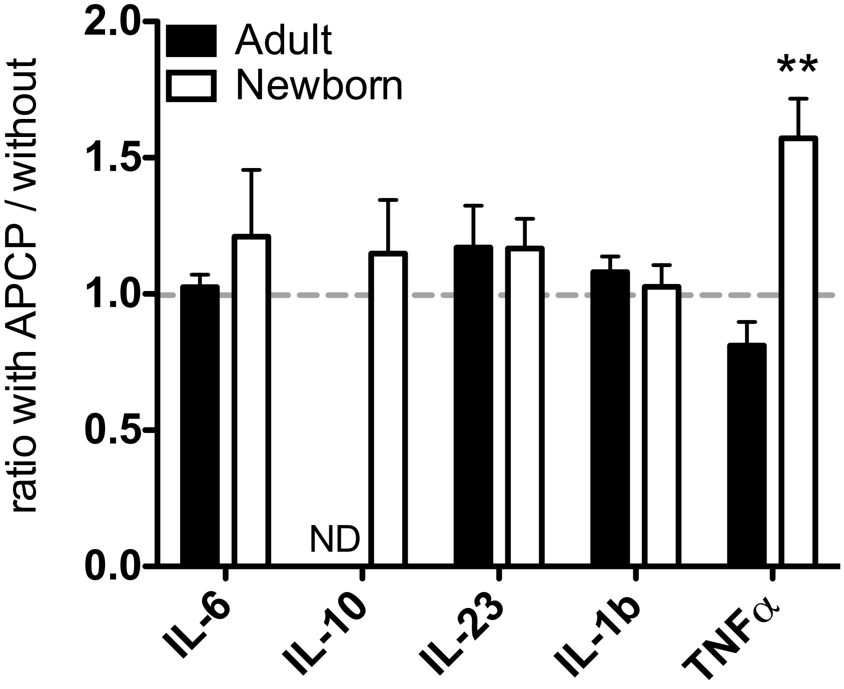
2.0
1.5
1.0
0.5
0.0
ratio with APCP / without
Adult
Newborn
**
ND
IL-6
IL-10
IL-23
IL-1b
TNFα
